# Statistical analysis plan of the study titled “A deprescribing programme aimed to optimize blood glucose-lowering medication in older people with type 2 diabetes mellitus — the OMED2 study: a randomized controlled trial”

**DOI:** 10.1186/s13063-026-09442-8

**Published:** 2026-01-30

**Authors:** Charlotte Andriessen, Peter P. Harms, Marieke T. Blom, Anna W. de Boer, G. Ardine de Wit, Ron Herings, Rob J. van Marum, Jacqueline G. Hugtenburg, Daniël van Raalte, Liselotte van Bloemendaal, Giel Nijpels, Rimke C. Vos, Petra Denig, Petra J. M. Elders

**Affiliations:** 1 Department of General Practice, Amsterdam UMC, Vrije Universiteit, Amsterdam, the Netherlands; 2 Amsterdam Public Health Research Institute, Amsterdam, the Netherlands; 3 Department of Public Health and Primary Care/Health Campus The Hague, LUMC, Leiden, The Netherlands; 4 Department of Health Sciences, Faculty of Beta Sciences, Vrije Universiteit Amsterdam, Amsterdam, the Netherlands; 5 Centre for Public Health, Healthcare and Society, National Institute of Public Health and the Environment, Bilthoven, the Netherlands; 6 PHARMO Institute for Drug Outcomes Studies, Utrecht, The Netherlands; 7 Department of Geriatric Medicine, Jeroen Bosch Hospital, ‘s-Hertogenbosch, the Netherlands; 8 Department of Clinical Pharmacology, Jeroen Bosch Hospital, ‘s-Hertogenbosch, the Netherlands; 9 Department of Elderly Care Medicine, Amsterdam University Medical Center, Amsterdam, the Netherlands; 10 Department of General Practice, Julius Center for Health Sciences and Primary Care, University Medical Center Utrecht, Utrecht University, Utrecht, the Netherlands; 11 Department of Internal Medicine, Diabetes Center, Amsterdam University Medical Center, Amsterdam, the Netherlands; 12 Department of Internal Medicine — Geriatrics, Amsterdam University Medical Center, Amsterdam, the Netherlands; 13 Department of Clinical Pharmacy and Pharmacology, University Medical Center Groningen, University of Groningen, Groningen, Netherlands

## Abstract

**Background:**

The OMED2 (*Optimization of Medication in Elderly with Diabetes*) study addresses the effect and implementation of integrating a deprescribing programme (DPP) in general practice. The aim of the DPP is to reduce glucose-lowering medication (SU/insulin) in overtreated older patients. The protocol for this study has been published previously. This statistical analysis plan (SAP) contains a more elaborate outline of the (statistical) methods we plan to use for data analysis.

**Methods:**

The OMED2 study is a randomized mixed-methods study with a 2-year follow-up period that compares the effect of the implementation of a DPP in general practice to regular care (control). In this SAP, we report on the (statistical) approaches that we plan to use to address the study objectives. The main objective of the OMED2 study is to examine the effect of the implementation of the DPP on diabetes complications, whereby the total number of diabetes complications related to undertreatment and overtreatment will be summed. Generalized linear mixed models with a Poisson distribution and the DPP as the main determinant will be used to test whether the total number of diabetes complications occurring from the start of the 2-year follow-up until the end of follow-up differs between intervention and control. The incident rate of the number of diabetes complications will be calculated to correct for possible differences in follow-up duration. The model will also include a random effect variable to allow for possible clustering effects by general practice. We will perform intention-to-treat analyses, which include all patients eligible for deprescribing, as well as per protocol analyses, which omit patients who were not deprescribed in the intervention arm. Additionally, approaches to study the implementation of the DPP and the cost-effectiveness of the implementation are outlined in the SAP.

**Trial registration:**

ISRCTN Registry ISRCTN50008265. Registered on 1 November 2024.

## Introduction

### Background and rationale

In the Netherlands, many elderly patients with type 2 diabetes (T2D) are at risk of hypoglycaemic episodes because they receive more glucose-lowering medication (i.e. insulin and/or sulphonyl derivatives) than recommended by the guideline of the Dutch College of General Practitioners [3]. Overtreatment with glucose-lowering medication can result in falling, hospitalization, and coma. Nevertheless, deprescribing of glucose-lowering medication in older patients is not widely adopted in general practice. One important reason that Dutch health care professionals (HCPs) are reluctant to deprescribing is that robust, scientific evidence on the safety of deprescribing glucose-lowering medication in elderly T2D patients is lacking.

The OMED2 study (*Optimization of Medication in Elderly with Diabetes*) is a randomized controlled trial to promote deprescribing glucose-lowering medication in general practice by means of a deprescribing programme (DPP). The main objectives of the OMED2 study are focused on the implementation of the DPP in general practice as well as the effect of deprescribing glucose-lowering medication and thereby increasing HbA1c levels on T2D complications. 

This SAP only concerns the data analyses to analyse the effect of the DPP intervention as described in the study design paper. The study design paper entails a more detailed description of the study design of the OMED2 study [1]. For readability, the research objectives on the implementation of the DPP have been separated from the research questions on the effect of the DPP as these use different methods of analyses. This statistical analysis plan (SAP) was based on the guidelines from Gamble et al. [6] and Stevens et al. [17].

## Objectives

Underscored concepts are explained more elaborately in Section “Variable definitions”.

### Primary study objective

To assess the effect of the DPP on the total number of *diabetes complications* related to under- and overtreatment combined compared to usual care (control). To address this objective, the total number of *diabetes complications* that are registered between the *start of the follow-up* period and the *end of the follow-up* period are summed. To address potential differences in follow-up duration, an incident rate is calculated for the outcome variable.

### Secondary study objectives

#### Related to diabetes complications

1. To assess the effect of the DPP on the total number of *diabetes complications* related to undertreatment compared to usual care (control). To address this objective, the total number of *diabetes complications* related to undertreatment that are registered between the *start of the follow-up* period and the *end of the follow-up* period is summed. To address potential differences in follow-up duration, an incident rate is calculated for the outcome variable.
2. To assess the effect of the DPP on the total number of *diabetes complications* related to undertreatment compared to usual care (control). To address this objective, the total number of *diabetes complications* related to undertreatment that are registered between the *start of the follow-up* period and the *end of the follow-up* period is summed. To address potential differences in follow-up duration, an incident rate is calculated for the outcome variable.

#### Research objectives on physical health outcomes

3. To assess the effect of the DPP vs. usual care (control) on the following health biomarkers: HbA1c (mmol/mol), fasting plasma glucose (mmol/l), systolic blood pressure (mmHg), and eGFR CKD-EPI (per 1.73 m^2^). *Baseline measurements* of health biomarkers will be compared with *measurements at 1 year of follow-up* and with *measurements at the end of follow-up*.

Research objectives on the implementation of the DPP will be discussed in “Part 2: Implementation analyses of OMED2-study” (page 17).

#### Research objective on changes in medication

4. Assess the use of diabetes medication at baseline, after 1 year of follow-up, and after 2 years of follow-up for both the intervention and control group. Both the medication dosage and amount of medication will be reported.

### Study methods

#### Trial design

This study entails a cluster randomized-controlled parallel-arm trial, wherein the effect of the DPP is compared to usual care during a 2-year follow-up period. The study is conducted by two research centres, i.e. Leiden University Medical Centre and Amsterdam University Medical Centre.

#### Randomization

Cluster randomization is performed on the level of GPs and PNs, stratified by practice size.

#### Sample size

Sample size calculation is described in the study design paper ([1], Trials). It was calculated that 406 patients and 86 practices need to be included to have sufficient power to analyse the primary outcome (i.e. total number of *diabetes complications*).

#### Framework

For the main outcome, a non-inferiority hypothesis testing framework will be used with a non-inferiority margin of 0.1 to assess whether the implementation of the DPP results in a similar total number of *diabetes complications* as compared to regular care (control). The non-inferiority margin means that the mean sum of complications should not differ by more than 0.1 complications between the intervention and control. If the difference between the mean sum of complications of the intervention and control falls within 0.1, we can conclude that the implementation of the DPP is non-inferior to regular care.

#### Statistical interim analyses and stopping guidance

Due to the nature of this study, i.e. the implementation of an already existing and approved guideline, no interim analyses are planned.

#### Timing of final analysis

**Fig. 1:**
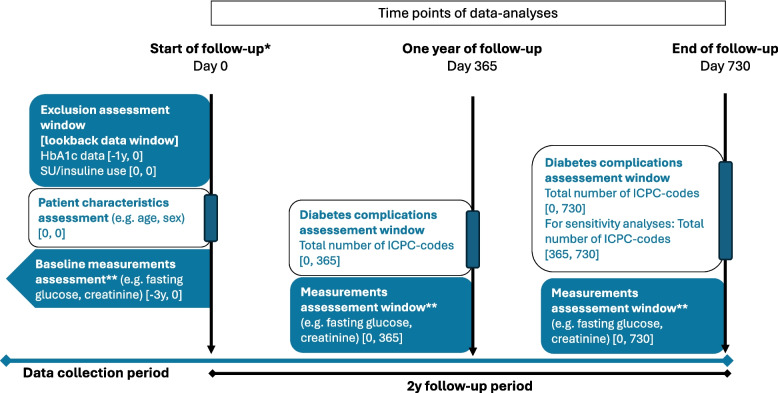
Study diagram of outcomes obtained in the OMED2 study. y, year; ICPC, International Classification of Primary Care. *Start of follow-up for intention-to-treat analyses is date of patient inclusion; start of follow-up for intervention group in per-protocol analyses is date of deprescribing. **Measurement closest prior to time point of data analyses is included

*Diabetes complications* will be counted from the *start of follow-up* until the *end of follow-up*, and the sum of these complications will be used for the primary analysis [12]. In addition, the difference in *diabetes complications* between the first year of follow-up compared to the second year of follow-up will be investigated. For this purpose, two groups of *diabetes complications* will be made. The first group will include the sum of *diabetes complications* that are registered from the *start of follow-up* until 1 year of follow-up (Fig. 1). The second group will include the sum of *diabetes complications* that are registered between 1 year of follow-up and the end of follow-up.

For the analysis of health biomarkers, *measurements at 1 year of follow-up* and *measurements at the end of follow-up* will be compared to *baseline measurements* (Fig. 1).

#### Timing of outcome assessments

Timing of outcome assessments is depicted in Table 1, which is derived from the study design paper [1]. Data from the electronic medical records (EMR) will be extracted after 12 months, 18 months, and 32 months after HCPs from the general practice have followed the educational session. These time points have been chosen so that patients who are identified at the second practice visit can have a 2-year follow-up period. It has also been taken into account that *deprescribing* of patients will not occur directly after the practice visit.

**Table 1:**
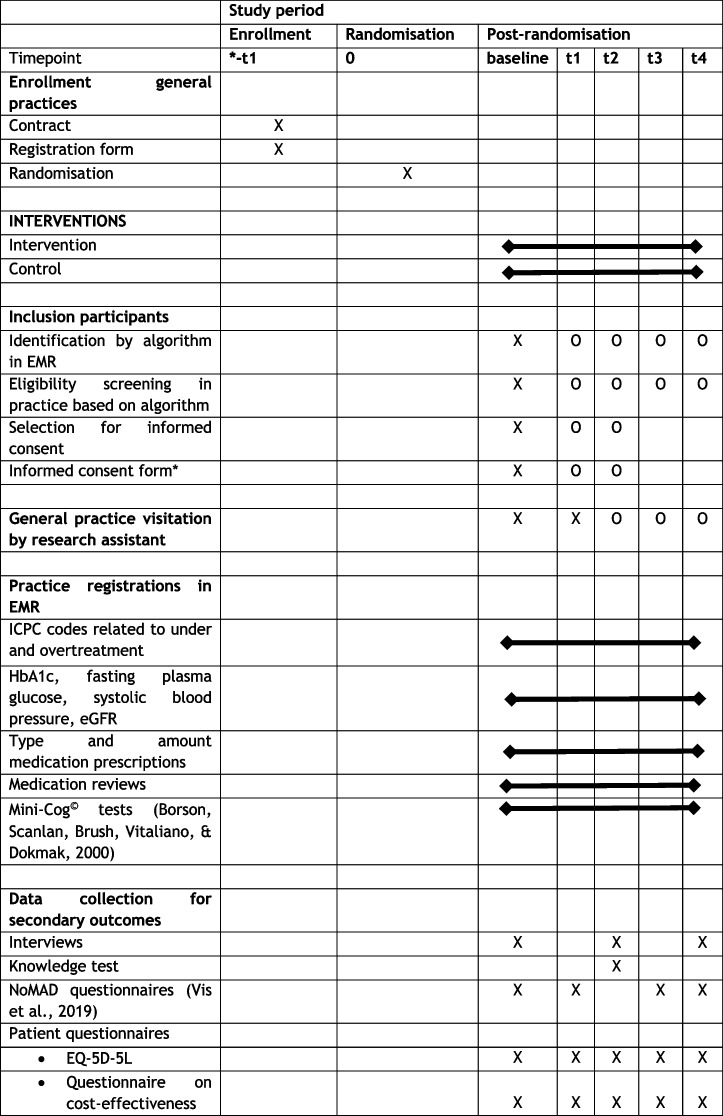
Standard Protocol Items: Recommendations for Interventional Trials (SPIRIT) diagram of the OMED2 study [4, 20]

*X*, event present at time point; *O*, event optional at time point. −t1, performed prior to randomization; baseline, study start, defined as the first day of the month in which the educational session occurred; t1, 6 months after baseline; t2, 1 year after baseline; t3, 1.5 years after baseline; t4, 2 years after baseline; *EMR*, electronic medical records; *ICPC*, International Classification of Primary Care; *eGFR*, estimated glomerular filtration rate; *NoMAD*, Normalization MeAsure Development

### Statistical principles

#### Confidence intervals and p-values

Results are considered significant if *p* < 0.05. Confidence intervals will be reported where appropriate and will be used to determine non-inferiority.

#### Adherence and protocol deviations

Adherence to the intervention is defined as the extent to which HCPs follow the DPP. The extent of the exposure is the size of the absolute reduction in daily dose of glucose-lowering medication (i.e. SU or insulin), which is discussed during the educational session. The deprescribing step needed depends on whether the patient uses SU or insulin and the type of insulin (long or short acting). We will summarize the initial changes in medication that have been performed. Barriers and facilitators for implementation of the DPP are also investigated. Implementation analyses of the OMED2-study are described in the second part of this SAP.

#### Analysis populations

The *intention-to-treat analysis* compares all patients eligible for deprescribing (see “Eligibility”), regardless of whether they were deprescribed or not, between the intervention and usual care group.

The *per-protocol analysis* compares patients who were deprescribed (excluding those who were restarted again afterwards) in the intervention group to patients eligible for deprescribing (regardless of whether deprescribing occurred) in the usual care group.

**Fig. 2:**
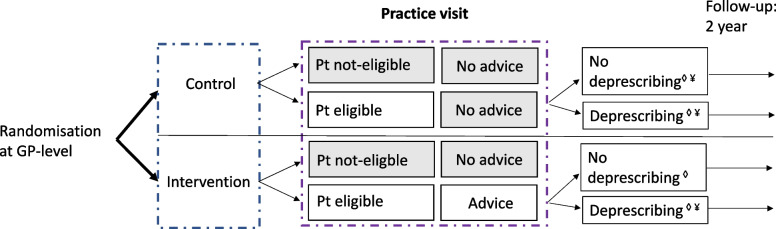
Study design of OMED2 study. ◊Sample included in intention-to-treat analysis. ¥Sample included in per-protocol analysis

For most research objectives, both an intention-to-treat analysis as well as a per-protocol analysis will be performed (Fig. 2).

*X*, event present at time point; *O*, event optional at time point. −t1, performed prior to randomization; baseline, study start, defined as the first day of the month in which the educational session occurred; t1, 6 months after baseline; t2, 1 year after baseline; t3, 1.5 years after baseline; t4, 2 years after baseline; *EMR*, electronic medical records; *ICPC*, International Classification of Primary Care; *eGFR*, estimated glomerular filtration rate; *NoMAD*, Normalization MeAsure Development

### Trial population

#### Screening data

To describe the representativeness of the trial sample, baseline characteristics of general practices as well as of patients will be reported. These data will be collected from the EMR.

#### Eligibility

Older patients (≥ 70 years) with T2D who are overtreated with SU derivatives and/or insulin are eligible to be included in the study. Overtreatment is defined as an HbA1c level < 54 mmol/mol, measured max. 1 year prior to the date of patient inclusion. Patients with HbA1c levels measured ≥ 1 year prior to the moment of patient inclusion are excluded from analysis. Patients are also excluded if regular diabetes care is not provided at the general practice. Whether a patient could be included in the study was discussed with the care provider at intervention practices, whereas in the control practices the researchers determined study inclusion based on care registration data.

Patients who did not use SU or insulin at the date of study inclusion (practice visit 1 or 2), or with an HbA1c level not below the target range, were not included in the study.

#### Recruitment

Recruitment of practices and patients is detailed in the study design paper.

#### Withdrawal/follow-up

It is anticipated that withdrawal from practices will be low, as the DPP intervention has been designed to limit the time investment for practices. If general practices withdraw from the intervention study, this will be reported, including the reason for withdrawal.

**Fig. 3:**
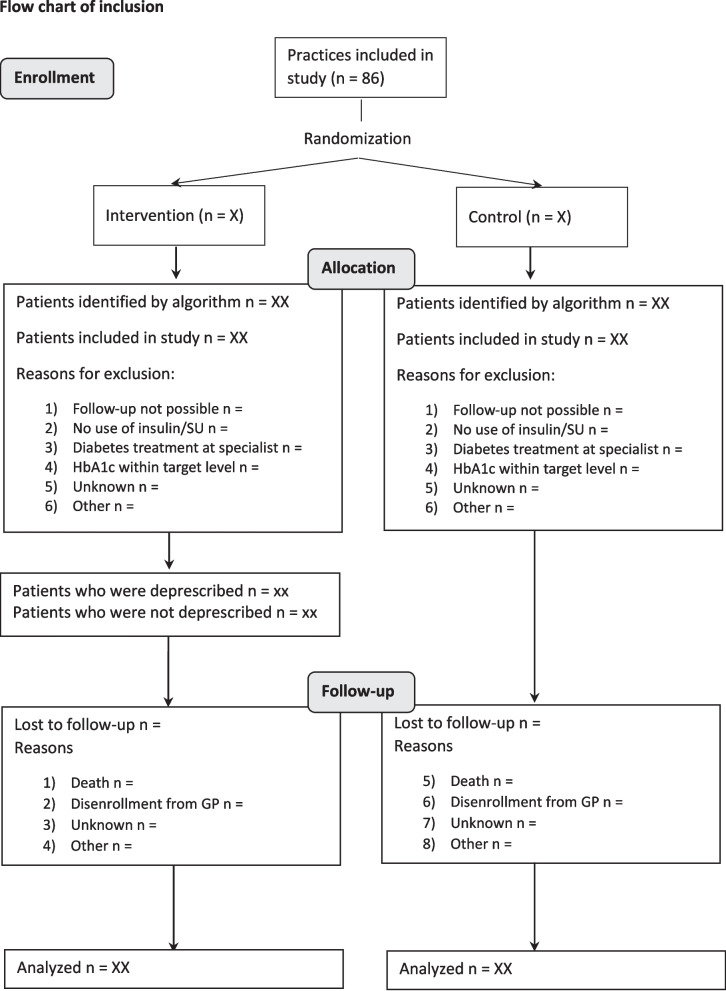
Flow diagram for the enrollment of patients in the study

Number of patients that are lost to follow–up will be reported, including reasons for lost to follow–up (Fig. 3). It will be assessed whether the group that has been lost to follow–up differs in baseline patient characteristics from the group that completed the follow–up period.

#### Baseline patient characteristics

Baseline characteristics will be presented using mean ± standard deviation for continuous, normally distributed variables and median ± interquartile range in case of non-normality (Table 2). Discrete variables will be presented as counts with percentages. Since this study entails regular care data, *baseline measurements* (e.g. HbA1c and BMI) were performed at different time points depending on at what date patients had planned their general practice visit. Definition of *baseline measurements* is therefore given in “Variable definitions”.

**Table 2:** Patient baseline characteristics

Patient characteristics	Total population *n* =	Intervention *n* = (%)	Control *n* = (%)
Age, years			
Women, *n* (%)			
Diabetes duration (% ≥ 10 years)			
Baseline HbA1c, mmol/mol			
BMI, kg/m^2^			
Kidney function^*^ (% poor)			
Multimorbidity^**^, *n*			
Polypharmacy^**^, *n* (% present)			
Medication, *n* (%)			
Metformin			
SU			
Insulin			
SGLT2i			
GLP1a			
DPP4			
Pioglitazone			

^*^Based on eGRF [13] and categorization of eGFR values in [8]. **Number of patients with multiple chronic diseases at study start; chronic diseases included the following: cardiovascular disease, alcohol abuse, smoking, airway diseases, obesity, diabetes, and kidney damage. ***Polypharmacy: use of ≥ 5 medications [9]

### Analysis

Prior to analysis, data needs to be extracted from the general practices, and a data quality control check will be performed. Information on these processes can be found at the end of the SAP. Variable of interest for this study are presented in Tables 3, 4, and 5.

**Table 3:** Outcome variables of the OMED2-study, how they are measured, and how they will be presented

Variable	Measurement of variable	Measurement unit	Type of variable	Presentation of variable
T2D complications	ICPC codes (EMR)	Count	Categorical	Count and percentage (?)
Complications of undertreatment	ICPC codes (EMR)	Count	Categorical	Count and percentage (?)
Complications of overtreatment	ICPC codes (EMR)	Count	Categorical	Count and percentage (?)
HbA1c	Blood draw (EMR)	Mmol/mol	Continuous	Mean ± st dev or median and IQR
Fasting plasma glucose	Blood draw (EMR)	Mmol/l	Continuous	Mean ± st dev or median and IQR
Systolic blood pressure	Blood pressure monitor (EMR)	mmHg	Continuous	Mean ± st dev or median and IQR
Kidney function	Blood draw, age, sex (EMR)	eGFR CKD-epi (per 1.73 m^2^)	Continuous	Mean ± st dev or median and IQR
Quality of life	EQ-5D-5L questionnaire	Utility score	Continuous	Mean ± st dev or median and IQR

**Table 4:** Variables that will be generated by transformation of existing variables

Variable	Calculation	Type of variable	Explanation
Time-window diabetes complications	Based on the date of the occurrence of diabetes complications (ICPC) relative to the start of follow-up	Categorical Complications in the first year of follow-up Complications in the second year of follow-up	Necessary to investigate the short- and long-term effects of the implementation of the DPP
eGFR	Calculated based on*eGFR using CKD-EPI (2021 update)*	Continuous	eGFR is not present in data extraction and is calculated using creatinine, sex, and age
Kidney function	Normal: *eGFR* > 60 Disturbed: *eGFR* 30–59 Poor: *eGFR* < 30	Categorical Normal Disturbed Poor	Categories of disturbed kidney function, based on literature
Per protocol	Patient in the intervention group is included when deprescribed (and not restarted) or if a patient is in the control group	Categorical Not included Included	Groups for per-protocol analysis
Time between patient identification and deprescribing	Date of deprescribing minus date of patient identification (i.e. practice visit 1 or 2)	Continuous (in days)	Estimation of how much time there is between patient identification and deprescribing
Time between baseline measurement and date of patient inclusion	Based on date of measurement and date of patient inclusion	Continuous (in days)	To gain insight in reliability of baseline measurements
Incident rate of diabetes complications	Total number of diabetes complications corrected for available follow-up period	Discrete	Necessary to correct total number of diabetes complications for possible differences in follow-up duration between participants

**Table 5:** Variables that need to be manually added to EMR data files

Variable	Type	Comment
Training	Date	Start date of general practice
Intervention_control	Categorical Intervention Control	As randomized at study start. Intervention group contained patients enrolled to practices that received the DPP and patients enrolled to control practices received regular care
Inclusion	Categorical Yes No	Inclusion established during practice visits and checked during data imputation by medicine student
Practice visit_Inclusion	Categorical First practice visit Second practice visit	Inclusion at first or second practice visit
Date_practice_visit_inclusion	Date	Date of practice visit at which patient was included in the study
Reason_of_exclusion	1. Categorical Unknown HbA1c not below reference values No use of sulphonylureas/insulin No regular DM treatment at general practice Age < 70	Reason of exclusion
Deprescribed	Categorical Yes No	Whether or not patient has been deprescibed
D_of_deprescribing	Date	Date of first deprescribing step
Reason_Not_Depr	Categorical Newly measured HbA1c not below target level Wish/fear of patient Decline patient Patient not visiting GP for a prolonged time DM care transferred to specialist Awaits new HbA1c Deceased Unknown	Reason for not deprescribing
Reference_values_HbA1c	Categorical 1. Unknown > 54 (for non-frail patients) > 59 (for frail patients) Do not determine HbA1c anymore	As indicated during practice visit and discussed with PN (only discussed in intervention arm)
SU_T0	Yes No	Use of SU at baseline
Insulin_T0	Yes No	Use of insulin at baseline
Metformin_T0	Yes No	Use of metformin at baseline
SGLT2i_T0	Yes No	Use of SGLT2i at baseline
GLP1a_T0	Yes No	Use of GLP1a at baseline
DPP4_T0	Yes No	Use of DPP4 at baseline
Pioglitazon_T0	Yes No	Use of pioglitazon at baseline
Med_Spec	Text	DM medication at T0 (including dose) written out
Med_deprescribed	Categorical SU Insulin SU + insulin None	Which medication has been deprescribed
Descr_deprescribing	Text	Description on deprescribing steps
Restart	Categorical Permanently deprescribed Medication restarted	Whether or not a patient has restarted medication after deprescribing. For the definition of restarted, see “Definition of variables”
Reason_restart	Categorical Unknown Complaints of hyper Fear patient Initiative of patient HbA1c above reference value Increasing glucose values	Reason why caregiver restarted SU/insulin after deprescribing, as deduced from the EMR
Date_of_restart	Date	Date at which SU/insulin has been restarted
Med_spec_restart	Text	Which medicine is restarted and at which dose

#### Variable definitions

- *Baseline measurements*: Measurements performed closest before the *start of follow-up*.
- *Cost-effectiveness analysis*: Evaluation of the difference in health effects when comparing the intervention with the control condition, relative to the difference in costs when comparing the two treatment arms. The health effects are expressed in quality-adjusted life years (see QALY definition), and the costs are total societal costs over the observation period (from inclusion to end of follow-up). The CEA is expressed in terms of the cost per QALY gained, when the intervention is compared to the control condition.
- *Deprescribing*: Lowering or discontinuing of glucose-lowering medication (i.e. insulin/SU derivates). If no recipe for a medication has been given out for a period of at least 6 months, the medication is considered to be discontinued. If short-acting insulin has been discontinued, but the dose of long-acting insulin has been increased, this does not count as deprescribing. If SU has been deprescribed but insulin has been started, this also does not count as deprescribing. Deprescribing is usually noted in the contact file by the health care provider; the date of this note is considered the date of deprescribing. If such a note is missing, the date of deprescribing will be estimated based on the median number of days between the date of patient identification and the date of deprescribing contained in the dataset.
- *Diabetes complications*: Registered in the EMR as International Classification of Primary Care (ICPC) codes. The incident ICPC codes relevant for this study have been established prior to the study start and are further described in the study design paper [1]. Newly identified journal ICPC codes that occur within the 2-year follow-up period are counted. In the case of missing journal ICPC codes, episode ICPC codes are counted. Patients can visit the general practice multiple times for the same diabetes complication, which could result in a misrepresentation of our results. To address this issue, ICPC codes are only counted if the interval between the first ICPC code and the second identical ICPC code exceeds a certain period of time (complaint-free period). The duration of the complaint-free period depends on the type of complication and is based on literature (Nielen, Davids, Gommer, Poos, & Verheij).
- *End of follow-up*: For the intention-to-treat analysis and the control group of the per-protocol analysis, this is the date which occurs 2 years after the date of patient inclusion (i.e. date of first or second practice visit). For the intervention group of the per-protocol analysis, this is the date which occurs 2 years after deprescribing has been initiated.
- *Intention-to-treat analysis*: Comparison between DPP intervention and control group of all patients who are eligible for deprescribing, regardless of whether or not patients have been deprescribed.
- *Lost–to–follow–up*: Patients who did not complete the 2–year follow–up period. Reasons for lost–to–follow–up include death, disenrollment from general practice, and drop–out of general practice. It is not always known why a patient has been lost to follow–up.
- *Measurements at 1 year of follow-up*: Measurements performed closest before 1 year of follow-up.
- *Measurements at end of follow-up*: Measurements performed closest before end of follow-up
- *Per-protocol analysis*: Comparison between intervention and control arm, only including patients who were deprescribed (and not restarted) in the intervention arm and including all eligible patients in the control arm.
- *Quality-adjusted life year (QALY)*: A full year in full health. This is the outcome measure of interest for a CEA. QALY assumes a multiplication of length of life (e.g. a full year) with quality of life. The quality of life is expressed on a 0 (dead) to 1 (full health) scale to allow multiplication.
- *Restarting medication after deprescribing*: If restarting medication after deprescribing results in an equal or higher dose of medication than the initial dose prior to deprescribing.
- *Start of follow-up*: For the intention-to-treat analysis and the control group of the per-protocol analysis, this is the date of patient inclusion (i.e. date of first or second practice visit). For the intervention group of the per-protocol analysis, this is the date at which deprescribing has been initiated.

## Analysis methods

### What analysis method will be used and how treatment effects will be presented

The primary outcome of this study is the total number of *diabetes complications* occurring from the *start of follow-up* until the *end of follow-up*, which is a 2-year period. The data will be analysed using generalized linear mixed models (GLMM) with a Poisson distribution, with DPP as the main determinant. To correct for possible differences in follow-up duration, for the outcome variable, the incident rate of the number of diabetes complications that occur during follow-up will be calculated. A random effect variable will be added to the GLMM model to allow for possible clustering effects by general practice. Sensitivity analyses will be performed to assess whether the total number of *diabetes complications* is different between the first year of follow-up and the second year of follow-up (see “Timing of final analysis”).

Linear mixed models will be used to assess the effect of the DPP on health biomarkers (e.g. HbA1c level). These models will contain a baseline outcome variable to account for the regression to the mean effect [18] and a random effect variable to allow for possible clustering effects by general practice. Furthermore, a variable for time will be included in the model to assess differences between *baseline measurements*, *measurements at 1 year of follow-up*, and *measurements at the end of follow-up*.

### Methods used for assumptions to be checked for statistical methods

Assumptions for the generalized linear mixed model that follows a Poisson distribution include the following:

1. The mean and variation of the distribution are the same.
  - Testing the model for overdispersion (i.e. if variation > mean) using the goodness-of-fit ratio of the Pearson χ^2^ statistic
  - If the goodness-of-fit ratio is > 2, overdispersion may be considered, and the negative binomial distribution will be used [14]
2. Relationship between numeric independent variables and log count of the outcome variable is linear
  - This will be checked using residual plots.

Assumptions for linear mixed models include the following:

1. Linear relationship between independent and dependent variable
  - Will be checked using a scatterplot on the *x*-axis the residuals and on the y-axis the outcome variable.
2. Normality of the residuals
  - A QQ-plot will be made.
3. Homoscedasticity (constant variance of residuals)
  - A scatterplot of residuals versus predicted values will be made.
4. No autocorrelation and no multicollinearity
  - The variance inflation factor (VIF) will be used to assess correlation between independent variables.

Linear mixed models tend to be relatively robust against violations of model assumptions. If any of the assumptions are violated, results will be interpreted with caution [16].

### Any planned subgroup analyses

In our pilot study, we observed that some patients were *restarting medication after deprescribing*. Subgroup analysis will be performed on this population to examine the extent to which they differ from the patients who did not restart their medication. We will do this by descriptively comparing characteristics of patients who restarted their medication after deprescribing with those who did not.

### Missing data

We will report the reasons why patients are lost to follow-up and will compare patients with deprescribing to those without deprescribing in the DPP intervention. We have selected analysis techniques that are relatively insensitive to missing data, and no imputation will be performed.

### Statistical software

IBM SPSS Statistics 28 (North Castle, New York, USA) will be used for statistical analyses.

## Part 2: implementation analyses of OMED2 study

*For the analyses of the implementation of the DPP, the same trial design and study population are used as described earlier (in *“Trial design”* section and *“Trial population”* section).*

### Research objectives on implementation of the DPP

*A mixed-methods data analysis will be performed to evaluate the public health impact of the implementation of the DPP in general practice, based on the RE-AIM model *[7]*. Topic lists and data analysis of interviews will be inspired by the frameworks by Reeve et al. *[15]* (patient interviews) and the Extended Normalization Process Theory (ENPT [HCP interviews]) *[10]*.*

## Re-AIM model (Reach, Efficacy, Adoption, Implementation, and Maintenance)

Table 6 depicts the data collection methods that are used to address all RE-AIM components.

**Table 6:** Quantitative and qualitive data collection methods involved in addressing the RE-AIM components

	**Reach**	**Effectiveness**	**Adoption**	**Implementation**	**Maintenance**
Study information	X				
EMR	X	X	X	X	
Interviews with HCPs	X	X	X	X	
Interviews with patients	X	X			
NoMAD questionnaire				X	
Knowledge questionnaire				X	
Focus group with HCPs	X	X			X

*EMR* electronic medical records, *HCPs* health care providers, *NoMAD* normalization measure development

- *Reach*: Who are affected by a programme, and does this population differ from the general population/those who do not receive the programme? Reach will be evaluated by the following:
  - Examining the absolute and relative number of frail elderly patients with diabetes included in the study and to what extent this group is representative for the total group of elderly patients with diabetes
  - In intervention practices, baseline characteristics of patients with deprescribing will be compared to characteristics of patients without deprescribing.

- *Effectiveness*: Determine the consequences and outcomes of the programme, which will be reported in the effect article and the cost-effectiveness article of the study.
- *Adoption*: The absolute number of included patients for whom deprescribing is initiated and who complete the total 2-year follow-up period. Knowledge transfer will be assessed with a knowledge questionnaire which will be sent to health care providers of both intervention and control practices after 1 year of follow-up.
- *Implementation*: Refers to the extent to which a programme is delivered as intended. Information on implementation can be derived from the interviews with care providers and patients. These will be analysed using the ENPT framework [10] and the framework from Reeve et al. [15], respectively. Additionally, components of the ENPT framework will be assessed semiquantitatively using scores from the Normalization MeAsure Development (NoMAD) questionnaire [20]. This instrument addresses coherence, cognitive participation, collective action, and reflexive monitoring to quantify the normalization of an intervention. The scores at the start of follow-up will be compared to those at the end of follow-up. The integration of the DPP in general practice care has been previously investigated in a feasibility study [2].
- *Maintenance*: Long-term maintenance of behaviour change. No quantitative measurements on the maintenance of the DPP have been performed at the end of the 2-year follow-up period. An additional focus group scheduled at the end of the follow-up will address maintenance of the DPP intervention.

## Part 3: cost-effectiveness analyses of OMED2 study

*For the analyses of the implementation of the DPP, the same trial design and study population are used as described earlier (in *“Trial design”* section and *“Trial population”* section).*

### Research objectives on the effect of deprescribing on cost-effectiveness

1. To assess the cost-effectiveness of the DPP, a cost-effectiveness analysis is performed based on patient questionnaires (sent out five times) on utilization of health care facilities and health status.

2. Amount and dose of blood glucose–lowering medication (i.e. SU and insulin) prescribed at baseline and changes in total daily medication use over the 2-year follow-up.

Definitions of *cost-effectiveness analysis* (CEA) and *QALY* are outlined in paragraph “Variable definitions”. The Guidelines for health economic evaluations version 2024 will be used for the cost-effectiveness evaluation [5].

The CEA of the DPP requires individual patient data both on the cost side and the effect side of the cost-effectiveness equation. Cost data will be based on the questionnaires of the patients who have given informed consent to receive questionnaires. The questionnaire assesses the nature and frequency of different types of formal (healthcare) and informal care received during the previous 3 months. Questionnaires will be sent at the start of the study and subsequently after every 6 months until the 2-year follow-up period has been reached. The questions which will be used to assess cost-effectiveness are based on the iMTA (Institute for Medical Technology Assessment) Medical Cost Questionnaire and reflect the types of healthcare and informal care that older diabetes patients typically use. Since the questionnaires are sent every 6 months, while they have a recall period of 3 months, it will be necessary to extrapolate the units of healthcare and informal care use during the 3-month periods that were not observed. This is done by linear extrapolation on an individual basis. This way, it will be possible to construct a profile of healthcare and informal care use over the entire follow-up period. For all patients, units of healthcare use (e.g. GP visits, drugs, inpatient and outpatient visits) and non-healthcare use (e.g. hours of informal care received) will be valued by applying the standard unit costs as communicated by the National Healthcare Institute in 2024 [11]. Total costs of the 2-year follow-up period will be added to arrive at individual cost estimates for all participants. Costs for the second year will be discounted using a discount rate of 3% as advised in Dutch guidelines for health economic evaluation. Two subcategories of costs will be summarized as well, i.e. healthcare costs and patient and family costs, the latter comprising of informal care. Mean costs will be calculated and compared between groups.

The outcome measure for the cost-effectiveness analysis is cost per QALY gained. The number of QALYs for every participant and the mean number of QALYs for both groups will be estimated using the EQ-5D-5L questionnaire [19]. This questionnaire produces cross-sectional health states of the participant, which will be transferred to utility scores with the Dutch “EQ-5D tariff”, based on societal valuations of these health states across the 0 (dead) to 1 (full health scale). These utility values will be used to calculate QALYs by combining utility scores and follow-up time (until end of study or dying). Quality of life will be measured at baseline and every 6 months until the 2-year follow-up period has been reached. QALYs will be estimated using an area under the curve approach: the mean of two consecutive 6-month utility scores will be corrected by a factor of 0.5 (reflecting a half-year period), and utility scores of four consecutive half-year periods will be added to calculate the total number of QALYs observed during a 2-year follow-up period.

Missing data for both costs and utilities will be handled using multiple imputation by chained equations (MICE), incorporating trial arm, cluster indicators, and baseline covariates into imputation models. We will generate 5–10 imputed datasets, depending on the level of missingness, to ensure stable estimates. Analyses for each dataset will be combined using Rubin’s rule.

For the final cost-effectiveness analysis, cost differences between groups will be divided by QALY differences between groups to estimate the cost per QALY gained. Analysis will be performed at the individual level, accounting for the clustered design. Costs and QALYs will be compared between arms using regression models adjusted for baseline covariates. For the latter, we will stick to the same covariates that are used for the primary effectiveness analysis, as it is important to align the analysis of effectiveness for both the clinical effectiveness and cost-effectiveness. Sampling uncertainty will be quantified using nonparametric bootstrapping at the cluster level. We will generate at least 5000 bootstrap replications to ensure stable estimation of confidence intervals around cost and QALY differences. Each bootstrap sample will be drawn with replacement at the cluster level to preserve the randomization structure. Cost-effectiveness planes will be plotted to visually inspect the distribution of estimates over the four quadrants of the plane. A cost-effectiveness acceptability curve (CEAC) will be drawn based on the 5000 bootstraps. The CEAC will show the probability that the intervention is cost-effective at different threshold levels for cost-effectiveness, e.g. the Dutch willingness to pay thresholds of 20,000 €, 50,0000 €, and 80,000 € per QALY. For each of these threshold values, we will express the probability that the DPP programme is cost-effective, relative to usual care given to older diabetes patients.

## Data Availability

Not applicable.

## Abbreviations

ANHA: Academisch Netwerk Huisartsen Amsterdam
CEA: Cost-effectiveness analysis
CEAC: Cost-effectiveness acceptability curve
DPP: Deprescribing programme
eGFR: Estimated glomerular filtration rate
EMR: Electronic medical record
ENPT: Extended Normalization Process Theory
EQ-5D-5L: EuroQol five-level
GLMM: Generalized linear mixed model
HCP: Health care professional
ICPC: International Classification of Primary Care
iMTA: Institute for Medical Technology Assessment
MICE: Multiple imputation by chained equations
NHG: Nederlands Huisartsen Genootschap
NoMAD: Normalization MeAsure Development
OMED2: Optimization of medication in elderly with diabetes
QALY: Quality-adjusted life year
RE-AIM: Reach, Efficacy, Adoption, Implementation, and Maintenance
SAP: Statistical analysis plan
SU: Sulphonyl urea
STIZON: Stichting Informatievoorziening voor Zorg en Onderzoek
T2D: Type 2 diabetes
